# The Impact of Aging in Acute Respiratory Distress Syndrome: A Clinical and Mechanistic Overview

**DOI:** 10.3389/fmed.2020.589553

**Published:** 2020-10-26

**Authors:** Ryan Brown, Michael C. McKelvey, Sinéad Ryan, Shannice Creane, Dermot Linden, Joseph C. Kidney, Daniel F. McAuley, Clifford C. Taggart, Sinéad Weldon

**Affiliations:** ^1^Airway Innate Immunity Research (AiiR) Group, Wellcome-Wolfson Institute for Experimental Medicine, School of Medicine, Dentistry and Biomedical Sciences, Queen's University Belfast, Belfast, United Kingdom; ^2^Department of Respiratory Medicine, Mater Hospital Belfast, Belfast, United Kingdom; ^3^Wellcome-Wolfson Institute for Experimental Medicine, School of Medicine, Dentistry and Biomedical Sciences, Queens University Belfast, Belfast, United Kingdom

**Keywords:** acute respiratory distress syndrome, acute lung injury, aging, immunosenescence, biomarkers

## Abstract

Acute respiratory distress syndrome (ARDS) is associated with increased morbidity and mortality in the elderly population (≥65 years of age). Additionally, age is widely reported as a risk factor for the development of ARDS. However, the underlying pathophysiological mechanisms behind the increased risk of developing, and increased severity of, ARDS in the elderly population are not fully understood. This is compounded by the significant heterogeneity observed in patients with ARDS. With an aging population worldwide, a better understanding of these mechanisms could facilitate the development of therapies to improve outcomes in this population. In this review, the current clinical evidence of age as a risk factor and prognostic indicator in ARDS and the potential underlying mechanisms that may contribute to these factors are outlined. In addition, research on age-dependent treatment options and biomarkers, as well as future prospects for targeting these underlying mechanisms, are discussed.

## Introduction

Acute respiratory distress syndrome (ARDS) is a common yet complex syndrome that develops in critically ill patients. Clinically, ARDS presents as acute hypoxemia and the presence of bilateral pulmonary infiltrates that cannot be fully ascribed to heart failure or fluid overload (1–3). This flooding of the alveoli with protein-rich edema is associated with the breakdown of the alveolar-capillary unit and, in many cases, the influx of neutrophils and other immune cells into the air spaces. These immune cells, along with activated epithelial and endothelial cells, release numerous pro-inflammatory mediators, which propagate a profound inflammatory response, and a battery of cytotoxic species, causing damage to the lung parenchyma (4). This endothelial and alveolar cell injury with fluid and cellular exudation has been termed as diffuse alveolar damage (DAD), however, data from biopsy and autopsy studies have shown that only half of those who meet the clinical definition of ARDS present with DAD (5–7). The presence of DAD is associated with increased mortality in ARDS (7). Despite over 50 years of investigation, ARDS is still under-recognized and no specific, effective treatments are available (8, 9). Mortality due to ARDS remains high, with most estimates indicating mortality rates in the region of 30–50% (8, 10, 11). ARDS is a heterogeneous disease process that may be triggered by a variety of direct or indirect pulmonary injuries including pneumonia, aspiration, non-protective mechanical ventilation, chest trauma, sepsis, and acute pancreatitis. Although ARDS can affect people of all ages, increasing age is a widely reported risk factor and is associated with increased mortality in ARDS patients (12, 13). This relationship has been emphasized by the current Covid-19 pandemic, in which severe acute respiratory syndrome coronavirus 2 (SARS-CoV-2), a causative agent of ARDS, has exhibited significant mortality among older patients (14–16).

The world's population is aging; over 65s are expected to account for 1 in 6 of the population by 2050, an increase from 1 in 11 in 2019 (17). With a strong association between aging and increased incidence of disease, including respiratory conditions such as chronic obstructive pulmonary disease and acute infection (18), a thorough understanding of age-related changes in the lung and how these impact on disease risk and severity will be important to effectively manage the health of an ever aging population. Age-associated structural changes in the lung mainly encompass increased alveolar spaces, reduced elasticity (19, 20) and a gradual decrease in lung function (21). An accumulation of senescent cells in the lung is observed in the elderly population (22). The release of soluble mediators by senescent cells contributes to persistent, low-level inflammation (23, 24), and impaired regenerative abilities (25). Similarly, the phenomenon of inflamm-aging is apparent in elderly cohorts where heightened levels of inflammatory factors are observed but several cell types including neutrophils and macrophages have attenuated phagocytic capabilities (26, 27). Thus, there are a number of pathophysiological features of the aging lung that may contribute to increased disease severity in elderly populations, which will be discussed in further detail in this review.

## Clinical Characteristics of Aging in ARDS

To date, few studies have focused primarily on assessing age *per se* as a risk factor for ARDS. Patients with ARDS often carry multiple risk factors and co-morbidities, some of which may be influenced by age themselves, which makes isolating the influence of age particularly challenging. Epidemiological studies frequently report the mean/median age of patients with ARDS at 55–65 years, making it a disease of late middle-age rather than exclusively old age (8, 10, 11, 28). One approach is to quantify the incidence of ARDS in the general population within different age groups. A study in the United States reported an overall incidence of 64 cases per 100,000 person-years but incidences of up to 306 cases per 100,000 person-years in the 75–84 years age group (10). Studies in Spain (29) and Taiwan (30) have shown similar age-dependent increases in incidence in the general population. The relationship between age and ARDS can also be examined by comparing cohorts of patients with another risk factor (pneumonia, trauma etc.) who did not develop ARDS with patients who had the same risk factor and did develop ARDS. Such analysis reveals that age appears to be a risk factor for ARDS in combination with certain other etiologies or risk factors. For instance, a large study of trauma patients by Johnston et al. demonstrated that the mean age of patients who developed ARDS was significantly higher than that of those who did not, and that the risk of developing ARDS increased up to 60–69 years of age (31). Likewise, patients with Covid-19 pneumonia who develop ARDS are significantly older than those who do not (16). However, in a recent study by Iriyama et al., there was no significant difference between the mean age of patients who developed ARDS following non-pulmonary sepsis and those who did not (32). Similarly, the age of patients who developed ARDS did not vary significantly from those who did not following out-of-hospital cardiac arrest (33), neutropenia after hematologic malignancy (34), or kidney transplant (35). Thus, it appears that while older age may generally be considered a risk factor for ARDS, larger studies are required to assess this relationship in ARDS with particular etiologies, especially indirect ARDS.

Accurately extrapolating the prognostic implication of age in ARDS is challenging. Observational data is derived from retrospective studies, which have inherent methodological flaws whilst elderly patients may frequently be excluded from randomized controlled trials. Consequently, the existing evidence-base may be prone to inaccuracy. Age is associated with worse ICU outcome and is embedded within several critical illness severity scoring systems, such as SAPS (simplified acute physiology score) and APACHE (acute physiology and chronic health evaluation) (36). These scores provide utility in prognostic prediction in the ICU setting, however, they lack specificity (37). There are currently no validated mortality prediction scoring systems in ARDS (36). Villar et al. prospectively evaluated a 9-point scoring tool (APPS) encompassing clinical parameters including age, PaO_2_/FIO_2_ and plateau pressure at 24 h following ARDS diagnosis (*n* = 600) (38). The authors stratified patients into three severity groups, assigning higher score in accordance with age group and reported a significant association with mortality (38). Subsequent external validation of the APPS score concluded that the model showed moderate-accuracy in predicting all-cause hospital mortality (39). Within adult populations, increased age is generally associated with higher disease severity (29). Several investigators have reported age as an independent predictor of mortality in ARDS (40, 41). It should be emphasized that substantial heterogeneity exists within the syndrome and non-linear associations between age and mortality have been reported within specific ARDS phenotypes [i.e., trauma; (42, 43)]. When considering the trauma-ARDS cohort, the highest burden of mortality has been shown to exist at the extremes of age (42, 43). A large retrospective review of a national trauma database (*n* = 1,297,190) reported the ARDS-related mortality was highest in those ≥80 and ≤4 years (42). Other investigators have reported no significant difference in mortality using a dichotomous age cut-off of 65 years, potentially supporting the notion that the relationship between age and ARDS may be more complex (44). While age is associated with ARDS mortality, there is no significant difference between ventilator or ICU free days, length of stay in ICU or length of stay in hospital between patients above 65 and those under 65 (12, 45). Indeed, a number of studies have concluded that co-morbidities rather than age primarily affect prognosis following ICU admission (46–48). Further elucidation of the impact of age upon prognosis in ARDS is required and well-designed prospective observational studies are paramount.

## Mechanisms of Age-Dependent Risk and Severity in ARDS

**Figure 1:**
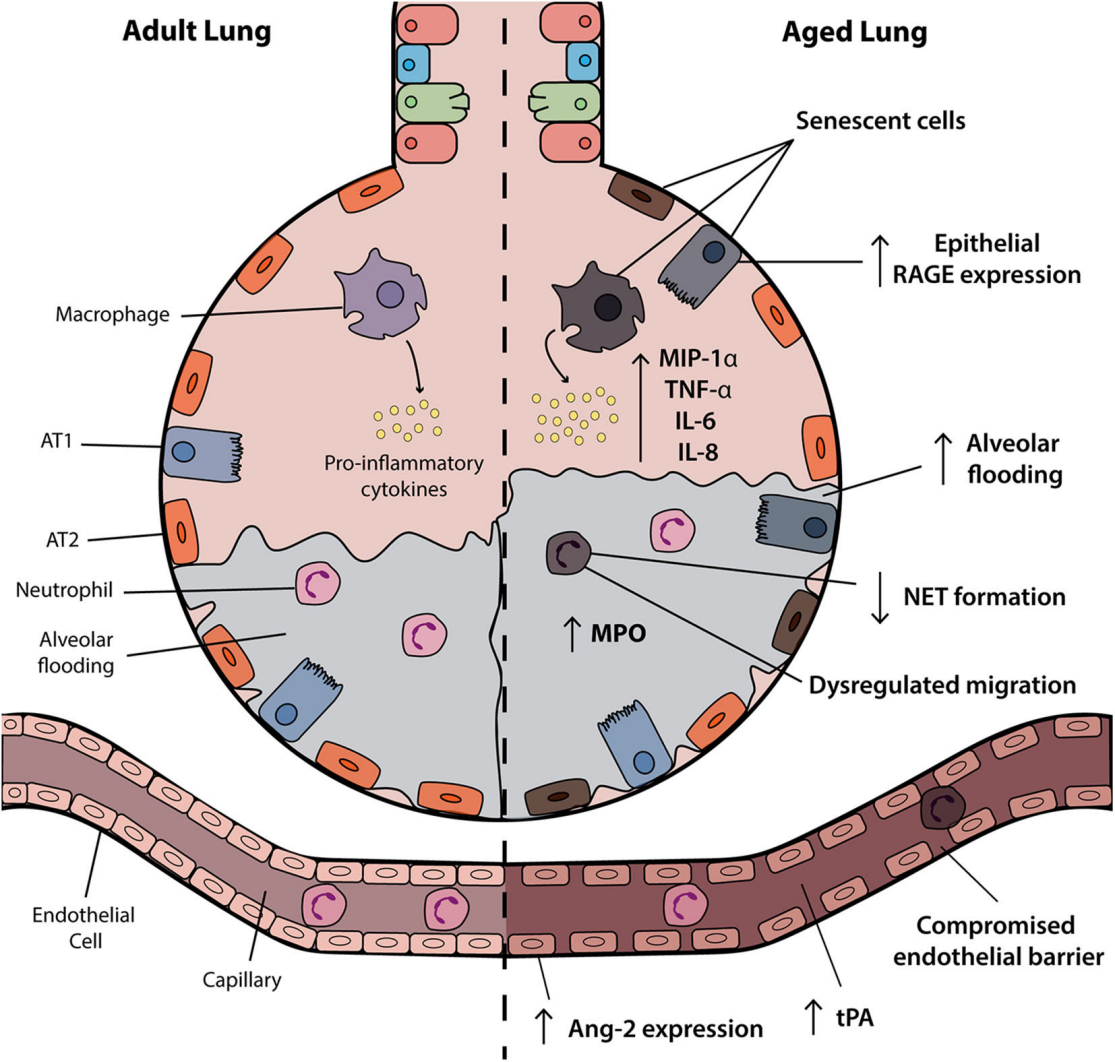
Age-associated changes in the ARDS lung. The aged alveolus in acute respiratory distress syndrome (ARDS) demonstrates a number of age-related changes in the alveolar epithelium, vasculature and immune cells that may contribute to disease pathogenesis in elderly patients. Vascular permeability may be affected by changes in angiopoietin-2 (Ang-2) expression and receptor for advanced glycation end products (RAGE) signaling is associated with inflammation and progression of the exudative and fibroproliferative phases of ARDS. Accumulation of senescent cells and the senescence-associated secretory phenotype (SASP) results in the release of soluble pro-inflammatory mediators such as IL-6 and IL-8. Although increased levels of inflammatory factors are observed, neutrophils and macrophages may have attenuated functional activities. AT1, alveolar type I; ATII, alveolar type II; NET, neutrophil extracellular trap; tPA, tissue plasminogen activator.

A number of factors influence disease severity in ARDS including features of the immune system, vasculature and structural components of the airway, as highlighted in Figure 1. Research to date has highlighted a number of mechanisms through which age may affect these systems and alter the risk of developing and/or disease severity in ARDS.

The decline in immune function with age is well-documented and is referred to as immunosenescence. Immune cell activation is a major mediator of inflammation in ARDS and immunosenescence may impact on the pathogenesis and outcomes in the aged ARDS subpopulation. Experimental murine models of ARDS induced by endotoxin indicate that aged mice display increased bronchoalveolar lavage fluid (BALF) cell counts, protein concentration, and cytokines such as the neutrophil chemoattractant CXCL1/KC in comparison to younger mice (49–51). Alterations in innate immune cell function, such as macrophage and neutrophils, may contribute to this heightened inflammatory response (52, 53). Alveolar macrophages (AMs) have increased expression of genes associated with lung injury and fibrosis (54) and defective phagocytic ability (26). In one study of lipopolysaccharide (LPS)-induced acute lung injury (ALI), expression of the M1-macrophage markers, CD80 and CD86, were increased in AMs from older mice and *in vitro* these cells had increased propensity to produce MIP-1α in response to LPS (50). Impairment of antigen presentation in aging macrophages has also been observed and resulting loss of bacterial and viral clearance from the lung could further promote inflammation and damage (55). In addition, aberrant neutrophil responses such as dysregulated chemotaxis (27) and the inability to form neutrophil extracellular traps (56) were observed in elderly cohorts. Studies across a breadth of ages in a small cohort of patients with ARDS suggest a correlation between age and the neutrophil biomarker myeloperoxidase (MPO) in BALF (57). Impaired neutrophil phagocytosis and microbial killing has also been observed in the elderly (58). The output of adaptive immune cells, particularly T cells, reduces over time. However, regulatory T (Treg) cells, which are responsible for regulation of immune responses, increased with age (50, 59). In experimental ARDS, aged groups show concomitant increases in both Tregs and inflammatory markers in comparison to younger groups (50). This suggests that while Treg numbers may be increased in the elderly, their anti-inflammatory function may be repressed. Clinically, there has been no consensus on the role of Tregs in ARDS with both protective (60, 61) and deleterious (62) effects observed. Furthermore, impaired adaptive immunity with decreased CD8^+^ T cells in advancing age may contribute to poor prognosis in ARDS (50). Recently published research brings together single cell sequencing data and tissue proteomics to document cell specific changes associated with aging (54, 63). Overall, changes in immune cell function may drive age related ARDS pathogenesis and further characterization of these changes may highlight potential targeted therapies that may be beneficial in an elderly population.

In addition to the immune system, age-related changes in structural components of the airway and vasculature may also contribute to ARDS pathogenesis. Within the vasculature, changes in the expression of angiopoietin-2 (Ang-2) have been linked to disease severity in ARDS (64). Ang-2 is a growth factor involved in angiogenesis which also plays a role in modulating endothelial permeability. Increased Ang-2 levels result in endothelial destabilization and increased vascular permeability (65). A positive correlation between Ang-2 and age has been observed in animal models and in the clinical setting (66, 67). Increases in Ang-2 may therefore be associated with ARDS severity in aging patients via increased vascular permeability and alveolar filling. Aging is also associated with an accumulation of senescent cells (68). Senescent endothelial cells (ECs) in ARDS demonstrate exacerbated permeability responses and this permeability was sustained by the upregulation of the reactive oxygen species (ROS)-generating enzyme NADPH oxidase-4 (Nox4) (69). In addition, Nox4 interacts with toll like receptor (TLR)-4 inducing NF-κB activation and inflammation (70). Taken together, these effects may contribute to increased ARDS severity, and inhibition of Nox-4 attenuated ALI in mice (71). Oxidative damage plays a significant role in age-related disease severity. Both aging and ARDS are associated with a cumulative oxidant burden and the generation of oxidized phospholipids has been linked to vascular leakage and inflammation and, as a result, the pathogenesis of ALI (72).

The structural components and mechanics of the lung also play an important role in airway pathophysiology. Aging is associated with changes in lung function and respiratory mechanics. Increased compliance and decreased small airway diameter in the elderly increase the severity of lung injury under mechanical ventilation (73). In addition to vascular ECs, senescence of airway epithelial cells and fibroblasts has also been linked to the pathogenesis of idiopathic pulmonary fibrosis through their expression of pro-inflammatory and profibrotic factors (74). This may also be of relevance in ARDS where a late fibrotic phase is associated with poor prognosis, high mortality and prolonged ventilator dependence (75). Targeting of senescent cells was beneficial in *in vivo* models of fibrosis, improving lung function and physical health (74). A decline in cellular autophagic function has also been observed in the aging lung (18) and induction of autophagy protected against lung permeability in models of ALI (76), highlighting the importance of this process. Another potential mechanism by which aging may contribute to increased ARDS severity is through the upregulation of receptor for advanced glycation end products (RAGE), a marker of type I alveolar epithelial cell injury (77). RAGE signaling likely promotes progression of both the exudative and fibroproliferative phases of ARDS (78, 79). Upregulation of RAGE was observed in mouse models of ALI and in ARDS patients and was associated with increased disease severity including increased mortality and fewer ventilator-free and organ failure-free days (77, 80). Treatment with recombinant soluble (s)RAGE, which acts as a decoy receptor, reduced inflammation in a murine model of LPS-induced ALI, suggesting that sRAGE participates in negative feedback after excessive inflammatory processes (81). Increased RAGE levels in the elderly may represent a potential mechanism through which aging effects ARDS severity, although a better understanding of how RAGE and its ligands impact pulmonary inflammation is needed (82).

## Impact of Age on Treatment Strategies and Specific Biomarkers

Biomarkers represent an important tool in diagnostics and patient stratification. However, to date, no single specific ARDS biomarker has been validated (83). Analysis of systemic inflammatory mediators and endothelial activation markers in plasma from adults with ARDS found that, compared to younger patients, elderly patients had lower levels of inflammatory mediators and endothelial activation markers (interleukin (IL)-6, IL-8, IL-10, interferon-γ, fractalkine, intracellular adhesion molecule (ICAM)-1, E-selectin) and higher levels of platelet factor-4 and tissue plasminogen activator (tPA) (12). Furthermore, advanced age was found to be independently associated with increased plasma levels of tPA and decreased plasma levels of fractalkine and E-selectin (12). However, only tPA was linked with outcome, accounting for 10% of the association between age and outcome. Although these findings suggest a possible link between aging, fibrinolysis and mortality, further work is needed to determine the role of tPA. More recently, Schouten et al. analyzed age-dependent differences in inflammatory and endothelial activation markers in BALF from ARDS patients of various ages (57). Although no difference was detected between adult and elderly cohorts, higher levels of neutrophil markers were found with increasing age and the association between increasing age and increased levels of MPO, IL-10, P-selectin, and decreased ICAM-1 remained significant after correction for severity of ARDS (57). These data suggest that plasma may be more useful in identifying age-dependent differences in ARDS patients although longitudinal analysis of BALF and plasma from the same cohort of patients may be required to shed light on the pathophysiology of pulmonary vs. systemic responses and how they interact across the spectrum of ARDS phenotypes. Like other diseases, variations in published data highlight the difficulties in identifying biomarkers that relate to disease severity and outcome in this heterogeneous syndrome, and a broader “omic” (e.g., proteomic) approach may be required. The identification of specific biomarkers would be helpful to identify those in the elderly population most at risk and to guide potential treatment options, although this may be a difficult feat.

Conclusive evidence to support age-dependent differences in treatment responses is lacking. However, there is evidence from both *in vivo* (84, 85) and computational studies (73, 86) to suggest that ventilator induced lung injury (VILI) may be more prevalent in the elderly population as a result of increased lung compliance and stiffness. The use of conservative fluid management and low tidal volume ventilation (LTVV) has been shown to attenuate this age-associated increase in ventilator associated mortality *in vivo* (87). Clinically, the benefits of LTVV are uncertain and adherence among clinicians is low (88, 89). Further study into the clinical benefits of LTVV specifically in the elderly subpopulation would be valuable. Extracorporeal membrane oxygenation (ECMO) has become a valuable therapy to support recovery in ARDS. Data on the use of ECMO in the elderly is limited, with most data coming from retrospective studies. Current data suggests that age is not a contraindication for ECMO, rather, its use should be decided on a case by case basis (90–92). Potential predictors that should be considered before initiation of ECMO support include presence of cardiogenic shock, APACHE II, and SAPS II scores (93). The accumulation of senescent cells has been suggested as a mechanism for increased mortality in elderly ARDS populations. Therefore, the use of senolytic drugs may represent an interesting therapy to improve outcomes in elderly patients with ARDS. Though senolytic drugs have not yet been evaluated clinically in ARDS, they have demonstrated benefits in preclinical models of fibrosis (74, 94). In the future, the identification of those most at risk of developing ARDS, along with the identification of the most effective treatment strategies for phenotypically and demographically distinct groups of patients, will be of paramount importance in reducing mortality in the elderly ARDS population.

## Conclusions and Future Directions

Research to date has highlighted the significant analytical challenges that ARDS presents due to its complex etiology. Although aging may alter the immune system, vasculature, and airway epithelium to promote the pathogenesis of ARDS, further work is needed to elucidate these mechanisms and this will be critical to improving our understanding of the effects of aging on the risk of developing ARDS and its severity. While more remains to be learned, accumulation of senescent cells along with increased oxidative damage, defective autophagy, and increased vascular permeability due to age-related changes are likely to play a role. A better understanding of the contributions of these individual factors along with the potential impact of co-morbidities could help direct future therapeutic strategies. A clearer understanding of the treatment options that are most beneficial for elderly patients with ARDS will be vitally important to reduce mortality in this subpopulation. Cell therapies represent a promising therapeutic option in ARDS (95), and these may be of particular significance in the elderly population where senescence and immune-aging render host cells ineffective and the lungs exhibit age-related structural changes (26, 27, 69). Treatment with mesenchymal stem cells, endothelial progenitor cells, or Tregs may attenuate age-related pathology. Alternatively, slowing the process of aging to mitigate age-related functional decline may be worth exploring in ARDS. Indeed, calorie restriction slowed the rate of aging and reduced ARDS risk in a preclinical model (96). Other potential therapeutics targeting aging including rapamycin (97), mTOR inhibitors (98), and metformin (99) could also be examined in ARDS. These therapies have been discussed in a recent review (100) and represent a number of potential avenues for the development of more targeted therapies for elderly ARDS patients. The stratification of ARDS patients into hypoinflammatory and hyperinflammatory subphenotypes revolutionized the clinical perspective. Studies of several ARDS cohorts identified distinct subphenotypes based on plasma inflammatory markers, level of shock and metabolic acidosis (101–103). These subphenotypes appear to respond differently to treatment (102), and while age does not significantly differ between the subphenotypes in most of these cohorts (101, 103, 104), further studies focusing on the role of age as a determinant of ARDS subphenotypes may be a useful strategy to direct treatment. Alternatively, identification of subphenotypes within elderly ARDS patient populations allowing stratification of these patients may also prove useful.

## Author Contributions

All authors listed have made a substantial, direct and intellectual contribution to the work, and approved it for publication.

## Conflict of Interest

The authors declare that the research was conducted in the absence of any commercial or financial relationships that could be construed as a potential conflict of interest.
